# Supplementary material for: Heat adaptation of phage T7 under an extended genetic code
Source: Virus Evol. 2021 Dec 1;7(2):veab100. doi: 10.1093/ve/veab100 (PMC8923235; doi:10.1093/ve/veab100)
Supplement: veab100_Supp [file veab100_supp.zip › Sequencing_Analysis.zip!/Sequencing_Analysis/A1/evidence/RA_42.html]

BRESEQ :: Evidence


| Read alignment evidence... | | | | | | | | | | | |
| --- | --- | --- | --- | --- | --- | --- | --- | --- | --- | --- | --- |
|  | seq id | position | | ref | new | freq | score (cons/poly) | reads | annotation | genes | product |
| \* | NC\_001604.1 | 35,254 | 0 | A | G | 36.9% | 1733.4 / inf | 2801 | intergenic (–/–) | *–/–* | –/– |
| Reads supporting (aligned to +/- strand):  **ref** base A (963/783);  **new** base G (574/447);  **total** (1560/1241) | | | | | | | | | | | |
| Fisher's exact test for biased strand distribution *p*-value = 6.06e-01 | | | | | | | | | | | |
| Kolmogorov-Smirnov test that lower quality scores support variant *p*-value = 1.00e+00 | | | | | | | | | | | |

Only 100 of 2815 total aligned reads displayed.  

`GAAGAGGCCCGTGACCTCACTACGGATACTATCGGTGTCAATAACGATGGTCACTTGGATGCTCGTGGTCGTCGAATTGTGAACCTAGCGAACGCCGTGGATGACCGCGATGCTGTTCCGTTTGGTCAACTAAAGACCATGAACCAGAACTCATGGCAAGCACGTAATGAAGCCTTACAGTTCCGTAATGAGGCTGAGACTTTCAGAAACCAAGCGGAGGGCTTTAAGAACGAGTCCAGTACCAACGCTACGAACACAAAGCAGTGGCGCGATGAGACCAAGGGTTTCCGAGACGAAGCCAAGCGGTTCAAGAATACGGCTGGTCAATACGCTACATCTGCTGGGAACTCTGCTTCCGCTGCGCATCAATCTGAGGTAAACGCTGAGAACTCTGCCACAGCATCCGCTAACTCTGCTCATTTGGCAGAACAGCAAGCAGACCGTGCGGAACGTGAGGCAGACAAGCTGGAAAATTACAATGGATTGGCTGGTGCAATTGATAAGGTAGATGGAACCAATGTGTACTGGAAAGGAAATATTCACGCTAACGGGCGCCTTTACATGACCACAAACGGTTTTGACTGTGGCCAGTATCAACAG  >  NC_001604.1/34954‑35553`  

                                                                                                                                                                                                                                                                                                            `|`                                                                                                                                                                                                                                                                                                             
`gAAGAGGCCCGTGACCTCACTACGGATATTATCGGTGTCAATAACGATGGTCACTTGGATGCTCGTGGTCGTCGAATTGTGAACCTAGCGAACGCCGTGGATGACCGCGATGCTGTTCCGTTTTGTCAACTAAAGACCATGGACCAGAACTCATGGCAAGCACGTAATGAAGCCTTACAGTTCCGTAATGAGGCTGAGACTTTCAGAAACCAAGCGGAGGGCTTTAAGAACGAGTCCAGTACCAACGCTACGAAAACAAAGCAGAGGCGCGATGAGACCAAGGGTTTCCGAGACGAAGCCa                                                                                                                                                                                                                                                                                                             >  1:441171/1‑301 (MQ=255)`  
`gAAGAGGCCCGTGACCTCACTACGGATATTATCGGTGTCAATAACGATGGTCACTTGGATGCTCGTGGTCGTCGAATTGTGAACCTAGCGAACGCCGTGGATGACCGCGATGCTGTTCCGTTTGGTCAACTAACGACCATGTACCAGAACTCATGGCGAGCACGTTATGAAGCCTTACAGTTCCGTAATGAGGCTGAGACTTTCAGAAACCAAGCGGAGGGCTTTAAGAATGAGTCCAGTAACAACGCTACGAACACAAAGCAGTTGCGCGATGAGCAAAAGGGTTTCCGAGACGAAGCCa                                                                                                                                                                                                                                                                                                             >  1:272344/1‑301 (MQ=255)`  
     `ggCCCGTGACCTCACGACGGATATTATCGGTGTCAATAACGATGGTCACTTGGATGCTCGGGGTCGTCGAATTGTGAACCTAGCGAACGCCGTGGATGACCGCGATGCTGGTCCGTTTGGTCAACTAAAGACCATGGACCAGAACTCATGGCAAGCACGTAATGAAGCCTTACAGTTCCGTAATGAGGCTGAGACTTTCAGAAACCAATCGGAGGGCTTTAAGAACGAGTCCAGTACCAACGCTACGAACACAAAGCAGTGGCGCGATGAGACCAAGGGTTTCCGAGACGAAGCCAAGCgg                                                                                                                                                                                                                                                                                                        <  1:133736/301‑1 (MQ=255)`  
           `tGACCTCACTACGGATATTATCGGTGTCAATAACGATGGTCACTTGGATGCTCGTGGTCGTCGAATTGTGAACCTAGCGAACGCCGTGGATGACCGCGATGCTGTTCCGTTTGGTCAACTAAAGACCATGGACCATAACTCATGGCAAGCACGTAATGAAGCCTTACAGTTCCGTAATGAGGCTGAGACTTTCAGAAACCAAGCGGAGGGCTTTAAGAACGAGTCCAGTACCAACGCTACGAACACAAAGCAGTGGCGCGATGAGACCAAGGGTTTCCGAGACGAAGCCAAGCGGTTCAAg                                                                                                                                                                                                                                                                                                  >  1:200173/1‑301 (MQ=255)`  
           `tGACCTCACTACGGATATTATCGGTGTCAATAACGATGGTCACTTGGATGCTCGTGGTCGTCGAATTGTGAACCTAGCGAACGCCGTGGATGACCGCGATGCTGTTCCGTTTGGTCAACTAAAGACCATGGACCAGAACTCATGGCAAGCACGTAATGAAGCCTTACAGTTCCGTAATGAGGCTGAGACTTTCAGAAACCAAGCGGAGGGCTTTAAGAACGAGTCCAGTACCAACGCTACGAACACAAAGCAGTGGCGCGATGAGACCAAGGGTTTCCGAGACGAAGCCAAGCGGTTCAAg                                                                                                                                                                                                                                                                                                  <  1:423364/301‑1 (MQ=255)`  
            `gACCTAACTACGGATATTATCGGTGTCAATAACGATGGTCACTTGGATGCTCGTGGTCGTCGAATTGTGAACCTAGCGAACGCCGTGGATGACCGCGATGCTGTTCCGTTTGGTCAACTAAAGACCATGGACCAGAACTCATGGCAAGCTCGTAATGAAGCCTTACAGTTCCGTAATGAGGCTGAGACTTTCAGAAACCAAGCGGAGGGCTTTAAGAACGAGTCCAGTACCAACGCTACGAACACAAAGCAGTGGCGCGATGAGACCAAGGGTTTCCGAGACGAAGCCAAGCGGTTCAAGa                                                                                                                                                                                                                                                                                                 <  1:7465/301‑1 (MQ=255)`  
             `agacagcTACGGATATTATCTGTGTAAATAACGTTGGTCACTTGGATTCTCGTGGTCGTCGAATTGGGAACCTAGCGAACGCGGTGGATGACCGCGATGCTGTTCCGTTTGGTAAACTAAAGACCATGGACCAGAACTCATGGCAAGCACGTAATGAAGCCTTACAGTTCCGTAATGAGGCTGAGACTTTCAGAAACCAAGCGGAGGGCTTTAAGAACGAGTCCAGTACCAACGCTACGAACACAAAGCAGTGGCGCGATGAGACCAAGGGTTTCCGAGACGAAGCCGAGCGGTTCAAGaa                                                                                                                                                                                                                                                                                                <  1:357673/295‑1 (MQ=255)`  
                `tCACTACGTATACTATCGGTGTCAATAACGATGGTCACTTGGATGCTCGTGGTCGTCGAATTGTGAACCTAGCGAACGCCGTGGATGACCGCGATGCTGTTCCGTTTGGTCAACTAAAGACCATGGACCAGAACTCATGGCAAGCACGTAATGAAGCCTTACAGTTCCGTAATGAGGCTGAGACTTTCAGAAACCAAGCGGAGGGCTTTAAGAACGAGTCCAGTACCAACGCTACGCACACCAAGCAGTGGCGCGATGAGACCAAGTGTTTCCGAGACGAAGCCGAGCGGTTCAAGAATAc                                                                                                                                                                                                                                                                                             >  1:393111/1‑301 (MQ=255)`  
                  `aCTACGGATATTATCGGGTTCAATAACGATGGTCACTTGGATGCTCGTGGTCGTCGAATTGTGAACCTAGCGCACGCCGTGGATGACCGCGATGCTGTTCCGTTTGGTCAACTAAAGACCATGGACCAGAACTCATGGCAAGCACGTAATGAAGCCTTACAGTTCCGTAATGAGGCTGAGACTTTCAGAAACCAAGCGGAGGGCTTTAAGAACGAGTCCAGTACCAACGCTACGAACACAAAGCAGTGGCGCGATGAGACCAAGGG‑TTCCGAGACGAAGCCAAGCGGTTCAAGAATACGGc                                                                                                                                                                                                                                                                                          <  1:61896/301‑1 (MQ=255)`  
                   `gacagtaTAGTATCGGTGTAAATAACGATGGTCACTTGGAGGCGCGTGGTCGTCTAATTGTGAACCTAGCGAACGCCGTGGATGACCGCGATGCTGTTCCGTTTGGTCAACTAAAGACCATGGACCAGAACTCATGGCAAGCACGTAATGAAGCCTTACAGTTCCGTAATGAGGCTGAGACTTTCAGAAACCAAGCGGAGGGCTTTAAGAACGAGTCCAGTACCAACGCTACGAACACAAAGCAGTGGCGCGATGAGACCAAGGGTTTCCGAGACGAAGCCGAGCGGTTCAAGAATACGGc                                                                                                                                                                                                                                                                                          <  1:86193/295‑1 (MQ=255)`  
                   `caaCGGATATTATCAGGGTCAAGAACGAGTGTCACTGGGATGCTCGTGGTCGGCGAAGTGTGAACCTAGCGAACGCCGGGGATGAACGCGATGCTGTTCCGTTGGGTCAACGAAAGACCATAGACCATAACTCATGGCAAGCACGTAATGAAGCCTGACAGTTCCGTAATGAGGCTGAGACGTTCAGAAACCAAGGGGAGGGCTTTAAGAACGAGTCCAGTACCAACTCTACGAACACAAAGCAGTGGCGCGATGAGACCAAGGGTTTCCGAGACGAAGCCGAGCGGTTCAAGAATATGGc                                                                                                                                                                                                                                                                                          <  1:113697/299‑1 (MQ=255)`  
                   `cTACGTATACTATCGGTGTCAATAACGATGGTCACTTGGATGCTCGTGGTCGTCGAATTGTGAACCTAGCGAACGCCGTGGATGACCGCGATGCTGTTCCGTTTGGTCAACTAAAGACCATGGACCAGAACTCATGGCAAGCACGTAATGAAGCCTTACAGTTCCGTAATGAGGCTGAGACTTTCAGAAACCAAGCGGAGGGCTTTAAGAACGAGTCCAGTACCAACGCTACGAACACCAAGCAGTGGCGCGATGAGACCAAGGGTTCCCGAGACGAAGCCAAGCGGTTCAAGAATATGGc                                                                                                                                                                                                                                                                                          >  1:194870/1‑301 (MQ=255)`  
                   `cTACGTATACTATCGGTGTCAATAACGATGGTCACTTGGATGCTCGTGGTCGTCGAATTGTGAACCTAGCGAACGCCGTGGATGACCGCGATGCTGTTCCGTTTGGTCAACTAAAGACCATGGACCAGAACTCATGGCAAGCACGTAATGAAGCCTTACAGTTCCGTAATGAGGCTGAGACTTTCAGAAACCAAGCGGAGGGCTTTAAGAACGAGTCCAGTACCAACGCTACGAACACAAAGCAGTGGCGCGATGAGACCAAGGGTTTCCGAGACGAAGCCAAGCGGTTCAAGAATACGGc                                                                                                                                                                                                                                                                                          >  1:172538/1‑301 (MQ=255)`  
                       `ggATATTATCGGTGTCAATAACGATGGTCACTTGGATGCTCGTGGTCGTCGAATTGTGAACCTAGCGAACGCCGTGGATGACCGCGATGCTGTTCCGTTTGGTCAACTAAAGACCATGGACCAGAACTCATGGCAAGCACGTAATGAAGCCTTACAGTTCCGTAATGAGGCTGAGACTTTCAGAAACCAAGCGGAGGGCTTTAAGAACGAGTCCAGTACCAACGCTACGAACACAAAGCAGTGGCGCGATGAGACCAAGGGTTTCCGAGACGAAGCCAAGAGGGTCAAGAATACGGCTGGt                                                                                                                                                                                                                                                                                      >  1:456201/1‑301 (MQ=255)`  
                       `ggATATTATCGGTGTCAATAACGATGGTCACTTGGATGCTCGTGGTCGTCGAATTGTGAACCTAGCGAACGCCGTGGATGACCGCGATGCTGTTCCGTTTGGTCAACTAAAGAAAATGGACCAGAACTCATGGCAAGCACGTAATGAAGCCTTACAGTTCCGTAATGAGGCTGAGACTTTCAGAAACCAAGCGGAGGGATTTAAGAACGAGTCCAGTACCACAGCTACGAACACAAAGCAGTGGCGCTATGAGAGAAAGGGCTTCCGAGACGAAGACAAGCGGTTCAAGAATACGGcctgg                                                                                                                                                                                                                                                                                      >  1:314547/1‑297 (MQ=255)`  
                        `ataagagacagtgtCAATAACGATGTTCACTTGGATGCTCGTGGTCGTCGAATTGTGAACCTAGCGAACTCCGTGGATGACCGCGATGCTGTTCCGTTTGGTCAACTAAAGACCATGGACCAGAACTCATGGCAAGCACGTAATGAAGCCTTACAGTTTCGTAATGAGGCTGAGACTTTCAGAAACCAAGCGGAGGGCTTTAAGAACGAGTCCAGTACCAACGCTACGAACACAAAGCAGTGGCGCGATGAGACCAAGGGTTTCCGAGACGAAGCCGAGCGGTTCAAGAATACGGCTGGTc                                                                                                                                                                                                                                                                                     <  1:142049/291‑1 (MQ=255)`  
                          `tattATCGGTGTCAATAACGATGGTCACTTGGATGCTCGTGGTCGTCGAATTGTGAACCTAGCGAACGCCGTGGATGACCGCGATGCTGTTCCGTTTGGTCAACTAAAGACCATGGACCAGAACTCATGGCAAGCACGTAATGAAGCCTTACAGTTCCGTAATGAGGCTGAGACTTTCAGAAACAAAGCGGAGGGCTTTAAGAACGAGTCCAGTACCAACGCTACGAACACAAAGCAGTGGCGCGAGGAGACCAGAGGTTTCCGAGACGAAGCCAAGCGGTTAAAgtactgtgctatataa                                                                                                                                                                                                                                                                                   >  1:415191/4‑286 (MQ=255)`  
                            `ttATTGGTGTCAATAACGATGGTCACTTGGATGCTCGTGGTCGTCGAATTGTGAACCTAGCGAACGCCGTGGATGACCGCGATGCTGTTCCGTTTTGTCAACTAAAGACCATGGACCAGAACTCATGGCAAGCACGTAATGAAGCCTTACAGTTACGTAATGAGGCTGAGACTTTCAGAAACCAAGCGGAGGGCTTTAAGAACGAGTCCAGTACCAACGCTACGAACACAAAGCAGTGGCGCGATGAGACCAAGGGTTTCCGAGACGCAGCCGAGCGGTTCAAGAATACGGCTggcagtct                                                                                                                                                                                                                                                                                 >  1:70528/2‑295 (MQ=255)`  
                             `tATCGGTGTCAATAACGATGGTCACTTGGATGCTCGTGGTCGTCGAATTGTGAACCTAGCGAACGCCGTGGATGACCGCGATGCTGTTCCGTTTGGTCAACTAAAGACCATGGACCAGAACTCATGGCAAGCACGTAATGAAGCCTTACAGTTCCGTAATGAGGCTGAGACTTTCAGAAACCAAGCGGAGGGCTTTAAGAACGAGTCCAGTACCAACGCTACGAACACAAAGCAGTGGCGCGATGAGACCAAGGGTTTCCGAGACGAAGCCAAGCGGTTCAAGAATACGGCTGGTCAATAc                                                                                                                                                                                                                                                                                <  1:28094/301‑1 (MQ=255)`  
                             `tATCGGTGTCAATAACGATGGTCACTGGGATGCTCGTTGTCGTCTAATTGTGAACCTGGCGAACGCCGTGGATGACCGCGATGCTGTTCCGTTTGGTCAACAAAAGACCATGGACCAGAACTCATGGCAAGCACGTAATGATGCCTTACAGTTCCGTAATGAGGCTGAGACTTTCAGAAACAAAGCGGAGGGCTTTAAGAACGAGTCCAGTACCAACGCTACGAACACAAAGCAGTGGCGCGATGAGACCAAGGGTTTCCGAGACGAAGCCAAGCGGTTCAAGAATACGGCTGGTCAATAc                                                                                                                                                                                                                                                                                <  1:78879/301‑1 (MQ=255)`  
                                  `gtgtCAATAACGATGGTCACTTGGATGCTCGTGGTCGTCGAATTGTGAGCCTAGCGAACGCCGTGGATGACCGCGATGCTGTTCCGTTTGGTCAACTAAAGACCATGGACCAGAACTCATGGCAAGCACGTAATGAAGCCTTACAGTTCCGTAATGAGGCTGAGAATTTCAGAAACCAAGCGGAGGGCTTTAAGAACGAGTCCAGTACCAACGCTACGAACACAAAGCAGTGGCGCGATGAGACCAAGGGTTTCCGAGACGAACCCGAGCGGCTCAAGAATACGGCTGGTCAATACGCTAc                                                                                                                                                                                                                                                                           >  1:16337/1‑301 (MQ=255)`  
                                  `gtgtCAATAACGATGGTCACTGGGATGCTCGTGGTCGTCGAATTGTGAACCTAGCGAACGCCGTGGATGACCGCGATGCTGTTCCGTTTGGTCAACTAAAGACCATGGACCAGAACTCAGGGCAAGCACGTAATGAAGCCTTACAGTTCCGTAATGAGGCTGAGACTTTCAGAAACCAAGCGGAGGGCTTTAAGAACGAGTCCAGTACCAACGCTACGAACACAAAGCAGTGGCGCGATGAGACCAAGGGTTTCCGAGACGAAGCCAAGCGGTTCAAGGATACGGCTGGTCAATACGCTAc                                                                                                                                                                                                                                                                           <  1:479406/301‑1 (MQ=255)`  
                                  `gtgtCAATAACGATAGTCACTTGGATGCTCGTGGTCGTCGAATTGTGAAACTAGCGAACGCCGTGGATGACCGCGATGCTGTTCCGTTTGGTCAACTAAAGACCATGGACCAGAACTCATGGCAAGCACGTAATGAAGCCTTACAGTTCCGTAATGAGGATGAGACTTTCAGAAACCAAGCGGAGGGCTTTAAGAACGAGTCCAGTACCAACGCTACGAACACAAAGCAGTGGCGCGATGAGACCAAGGGTTTCCGAGACGAAGCCGAGCGGTTCAAGAATACGGCTGGTCAAAACACTAc                                                                                                                                                                                                                                                                           <  1:9045/301‑1 (MQ=255)`  
                                    `gtCAATAACGATGGTCACTTGGATGCTCGTGGTCGTCGAATTGTGAACCTATCGAACGCCGTGGATGACCGCGATGCTGTTCCGTTTGGTCAACTAAAGACCATTGACCAGAACTCATGGCAAGCACGTAATGAAGCCTTACAGTTCCGTAATGAGGCTGAGACTTTCAGAAACCAAGCGGAGGGCTTTAAGAACGAGTCCAGTACCAACGCTACGAACACAAAGCAGTGGCGCGATGAGACCAAGGGTTTCCGAGACGAAGCCAAGCGGGTCAAGaactgtctcttatacacatctccga                                                                                                                                                                                                                                                                         >  1:361850/1‑278 (MQ=255)`  
                                     `tataagagacaggTCACTTGGATGCTCGTGGTCGTCGAATTGTGAACCTAGCGAACGCCGTGGATGACCGCGATGCTGTTCCGTTTGGTCAACTAAAGACCATGGACCAGAACTCATGGCAAGCACGTAATGAATCCTTACAGTTCCGTAATGAGGCTGAGACTTTCAGAAACCAAGCGGAGGGCTTTAAGAACGAGTCCAGTACAAACGCTACGAACACAAAGCAGTGGCGCGATGAGACCAAGGGTTTCCGAGACGAAGCCAAGCGGTTCAAGAATACGGCTGGTCAATACGCTACATc                                                                                                                                                                                                                                                                        <  1:57833/290‑1 (MQ=255)`  
                                     `tCAATAACGATGGTCACTTGTAGGCTCGTGGTCGTCGAATTGTGAACCTAGCGAACGCCGTGGATGACCGCGATGCTGTTCCGTTTGGTCAACTAAAGACCATGTACCAGAACTCATGGCAAGCACGTAATGAAGCCTTACAGTTCCGTAATGAGGCTGAGACTTTCAGAAACCAAGCGGAGGGCTTTAAGAACGAGTCCAGTACCAACGCTACGAACACAAAGCAGTGGCGCGATGAGACCAAGGGTTTCCGAGACGAAGCCAAGCGGTTCAAGAATACGGCTGGTCAATACGCTACATc                                                                                                                                                                                                                                                                        <  1:378072/301‑1 (MQ=255)`  
                                     `tCAATAACGATGGTCACTTGGATGCTCGTGGTCGTCGAATTGTGAACCTAGCGAACGCCGTGGATGACCGCGATGCTGTTCCGTTTGGTCAACTAAAGACCATGGACCAGAACTCATGGCAAGCACGTAATGAAGCCTTACAGTTCCGTAATGAGGCTGAGACTTTCAGAAACCAAGCGGAGGGCTTTAAGAACGAGTCCAGTACCAACGCTACGAACACAAAGCAGTGGCGCGATGAGACCAAGGGTTTCCGAGACGAAGCCGAGCGGTTCAAGAATACGGCTGGTCAATACGCTACATc                                                                                                                                                                                                                                                                        <  1:100000/301‑1 (MQ=255)`  
                                          `aaCGATGGTCACTTGGATGCTCGTGGTCGTCGAATTGTGAACCTAGCGAACGCCGTGGATGACCGCGATGCTGTTCCGTTTGGTCAACTAAAGACCATGGACCAGAACTCATGGCAAGCACGTAATGAAGCCTTACAGTTCCGTAATGAGGCTGAGACTTTCAGAAACCAAGCGGAGGGCTTTAAGAACGAGTCCAGTACCAACGCTACGAACACAAAGCAGTGGCGCGATGAGACCAAGGGTTTCCGAGACGAAGCCAAGCGGTTAAAGAATACGGCTGGTCAATACGCTACATctgctg                                                                                                                                                                                                                                                                   >  1:207776/1‑301 (MQ=255)`  
                                           `aCGATGGTCACTTGGATGCTCGTGGTCGTCGAATTGTGAACCTAGCGAACGCCGTGGCTGACCGCGATGCTGTTCCGTTTGGTCAACTAAAGACCATGGACCAGAACTCATGGCAAGCACGTAATGAAGCCTTACAGTTCCGTAATGAGGCTGAGACTTTCAGAAACCAAGCGGAGGGCTTTAAGAACGAGTCCAGTACCAACGCTACGAACACAAAGCAGTGGCGCGATGAGACCAAGGGTTTCCGAGACGAAGCCGAGCGGTTCCAGAATACGGcctgtctcttatacacatctccgag                                                                                                                                                                                                                                                                  >  1:146744/1‑277 (MQ=255)`  
                                            `cGATGGTCACTTGGATGCTCGTGGTCGTCGAATTGTGAACCTAGCGAACGCCGTGGATGACCACGATGCTGTTCCGTTTGGTCAACTAAAGACCATGGACCAGAACTCATGGCAAGCACGTAATGAAGCCTTACAGTTCCGTAATGAGGCTGAGACTTTCAGAAACCAAGCGGAGGGCTTTAAGAACGAGTCCAGTGCCAACGCTACGAACACAAAGCAGTGGCGCGATGAGACCAAGGGTTTCCGAGACGAAGCCGAGCGGTTCAAGAATACGGCTGGTCAATACGCTACATCTGCTggg                                                                                                                                                                                                                                                                 >  1:234449/1‑301 (MQ=255)`  
                                               `tGGTCACTTGGATGCTCGTGGTCGTCGAATTGTGAACCTAGCGAACGCCGTGGATGACCGCGATGCTGTTCCGTTTGGTCAACTAAAGACCATGGACCAGAACTAATGGCAAGCACGTAATGAAGCCTTACAGTTCCGTAATGAGGCTGAGACTTTCAGAAACCAAGCGGAGGGCTTTAAGAACGAGTCCAGTACCAACGCTACGAACACAAAGCAGTGGCGCGATGAGACCAAGGGTTTCCGAGACGAAGCCGAGCGGTTCAAGAATACGGCTGGTCAATACGCTACATCTGCTGGGAAc                                                                                                                                                                                                                                                              <  1:436599/301‑1 (MQ=255)`  
                                                `ggTCACTTGGATGCTCGTGGTCGTCGAATTGTGAACCTAGCTAACGCCGTGGATGACCGCGATGCTGTTCCGTTTGGTCAACTAAAGACCATGGACCAGAACTCATGGCAAGCACGTAATGAAGCCTTACAGTTCCGTAATGAGGCTGAGACTTTCAGAAACCAAGCGGAGGGCTTTAAGAACGAGTCCAGTACCAACGCTACGAACACAAAGCAGTGGCGCGATGAGACCAAGGGTTTCCGAGACGAAGCCAAGCGGTTCGAGAATACGGCTGGTCAATACGCTACATCTGCTGGGAAct                                                                                                                                                                                                                                                             >  1:445385/1‑301 (MQ=255)`  
                                                                    `gcgtcgAATTGTTAACCTAGCGAACGCCGTGGATGACCGCGATGCTGTTCCGTTTGGTCAACTAAAGACCATGGACCAGAACTCATGGCAAGCACGTAATGAAGCCTTACAGTTCCGTAATGAGGCTGAGACTTTCAGAAACCAAGCGGAGGGCTTTAAGAACGAGTCCAGTACCAACGCTACGAACACAAAGCAGTGGCGCGATGAGACCAAGGGTTTCCGAGACGAAGCCGAGCGGTTCAAGAATACGGCTGGTCAATACGCTACATCTGCTGGGAACTCTGCTTCCGATGCGCATCaa                                                                                                                                                                                                                                         <  1:269098/300‑1 (MQ=255)`  
                                                                      `gtcgAATTGTGAACCTAGCGAACGCCGTGGATGACCGCGATGCTGTTCCGTTTGGTCAACTAAAGACCATGGACCAGAACTCATGGCAAGCACGTAATGAAGCCTTACAGTTCCGTAATGAGGCTGAGACTTTCAGAGACCAAGCGGAGGGCTTTAAGAACGAATCCAGTACCAACGCTACGAACACAAAGCAGTGGCGCGATGAGACCAAGGGTTTCCGAGACGCAGCCAAGCGGTTCAAGAATATGGCTGGTCAATCCGCTACATCTGCTGGGaccctgtctcttatacacatctccga                                                                                                                                                                                                                                       >  1:243643/1‑276 (MQ=255)`  
                                                                      `gtcgAATCGGGAACCCAGCGAAGTCCGTGGATGACCGCGATGCTGTGCGTGTTGTTCAACTAACGACCATGGACCAGAACTCCTGGCAATGACGTAATGAAGCCTTACAGTTCCGTAATGAGGCTGAGACTTTCAGCAACCAAGCGGAGGGCTTTAAGAACTATTCCAGTCCCAACTCTACGAACACAAAGCAGTGGCGCGATGAGACCAAGGGTTTCCGAGACGAAGCTGAGCGGTTCAAGAATACTGCTGGTCAATACGCTACATCTGCTGGGAACTCTGCTTCCGCTGCGCATCAATc                                                                                                                                                                                                                                       <  1:508323/301‑1 (MQ=255)`  
                                                                           `aTTGTGAACCGAGCGAACGCCGTGGATGACCGCGATGCTGTTCCGTTTGGTCAACTAAAGACCATGGACCAGAACTCATGGCAAGCACGTAAGGAAGCCTTACAGTTCCGTAATGAGGCTGAGACTTTCAGAAACCAAGCGGAGGGCTTTAAGAACGAGTCCAGTACCAACGCTACGAACACAACGCAGTGGCGCGATGAGACCAAGGGTTTCCGAGACGAAGCCAAGCGGTTCAAGAATACGGCTGGTCAATACGCTACATCTGCTGGGAACTCTGCTTCCGCTGCGCATCAATCTGAgg                                                                                                                                                                                                                                  <  1:121368/301‑1 (MQ=255)`  
                                                                            `acaggAACCTAGCGAACGCCGTGGATGACCGCGATGCTGTTCCGTTTGGTCAACTAAAGATCATGGACCAGAACTCATGGCAAGCACGTAATGAAGCCTTACAGTTCCGTAATGAGGCTGAGACTTTCAGAAACCAAGCGGAGGGCTTTAAGAACGAGTCCAGTACCAACGCTACGAACACAAAGCAGTGGCGCGATGAGACCAAGGGTTTCCGAGACGAAGCCAAGCGGTTCAAGAATACGGCTGGTCAATACGCTACATCTGCTGGGAACTCTGCTTCCGCTGCGCATCAATCTGAGGt                                                                                                                                                                                                                                 <  1:25460/297‑1 (MQ=255)`  
                                                                             `tgtgAACCTAGCGAACGCCGTGAATGACCGCGATGCTGTTCCGTTTGGTCAACTAAAGACCATGGACCAGAACTCATGGCAAGCACGTAATGAAGCCTTACAGTTCCGTCATGAGGCTGAGACTTTACGAAACCAATCGGAGGGCTTTAAGAACGAGTCCAGTACCAACGCTACGAACACAAAGCAGTGGCGCGATGAGACCAAGGGTTTACGAGAAGAAACCAAGCGTTTCAAGACTACGGCACGACAATCCGCAACATATGCTGGGCACTATGCTTCCACTGCGCATCAATcgaatgta                                                                                                                                                                                                                                >  1:498362/1‑294 (MQ=255)`  
                                                                                  `cgtgggctcggagatgtgtataagagacagCTGTTCCGTTTGGTCAACTAAAGACCATGGACCAGAACTCATGGCAAGCACGTAATGAAGCCTTACAGTTCCGTAATGAGGCTGAGACTTTCAGAAACCAAGCGGAGGGCTTTAAGAACGAGTCCAGTACCAACGCTACGAACACAAAGCAGTGGCGCGATGAGACCAAGGGTTTCCGAGACGAAGCCGAGCGGTTCAAGAATACGGCTGGTCAATACGCTACATCTGCTGGGAACTCTGCTTCCGCTGCGCATCAATCTGAGGTAAACGc                                                                                                                                                                                                                           <  1:52465/272‑1 (MQ=255)`  
                                                                                  `aCCTAGCGAACGCCGTGGATGACCGAGATGCGGTTCCGATTGGTCAACTAAAGAACATGGACCAGAACTCATGGCAAGCACGTAATGAAGCCTTACAGTGCCGTAATGAGGCGGAGACGTTCAGAAACCAAGCGGAGGGCGTTAATAACGAGTCCAGTACCAACGCTACTAACACAAAGCAGTGGCGCGATGAGACCAAGGGTTTCCGAGACGAAGCCAAGCGGTTCAAGAATACGGCTGGTCAATACGCTACATCTGCTGGGAACTGTGCTTCCGCTGCGCATCAATCTGAGGTAAACGc                                                                                                                                                                                                                           <  1:212396/301‑1 (MQ=255)`  
                                                                                   `ccTAGCGAACGCCGTGGATGACCGCGATGCTGTTCCGTTTGGTCAACTAAAGACCATGGACCAGAACTCATGGCACGCACGTAATGAAGCCTTACAGTTCCGTAATGAGGCTGAGACTTTCAGAAACCAAGCGGAGGGCTTTAAGAACGAGTCCAGTACCAACGCTACGAACACAAAGCAGTGGCGCGATGAGACCAGGGGTTTCCGAGACGAAGCCAAGCGGTTCAAGAATACGGCTGGTCAATACGCTACATCTGCTGGGAACTCTGCTTCCGCTGCGCATCAATCTGAGGTAAACGCt                                                                                                                                                                                                                          >  1:128820/1‑301 (MQ=255)`  
                                                                                   `ccTAGCGAACGCCGTGGATGACCGCGATGCTGTTCCGTTTGGTCAACTAAAGACCATGGACCAGAACTCATGGCAAGCACGTAATGAAGCCTTACAGTTTCGTAATGAGGCTGAGACTTTCAGAAACCAAGCGGAGGGCATTAAGAACGAGTTCAGTACCAACGCTAAGAACAAACAGCAGTGGCGCGATGAGACTAAGGGGTTCCGAGCCGAAGACGAGCGGTTCAAGAATACGGCTGGGCAATACGCTAAATTTGCTGGGAAATTTGCTTCCGCGGCGCATCAATCTGAGGTAAACgtt                                                                                                                                                                                                                          >  1:67114/1‑299 (MQ=255)`  
                                                                                           `aCGCCGTGGATGACCGCGAAGCTGTTCCGTTTGGTCAACTAAAGACCATGGACCAGAACTAATGGCAAGCACGTAATGAAGCCTTACAGTTCCGTAATGAGGCTGAGACTTTCAGAAACCAAGCGGAGGGCTTTAAGAACGAGTCCAGTACCAACGCTACGAACACAAAGCAGTGGCGCGATGAGACCAAGGGTTTCCGAGACGAAGCCAAGCGGTTCAAGAATACGGCTGGTCAATACGCTACATCTGCTGGGAACTCTGCTTCCGCTGCGCATCAATCTGAGGTAAACGCTGAGAActc                                                                                                                                                                                                                  <  1:22212/301‑1 (MQ=255)`  
                                                                                              `ccGTGGATGACCGCGATGCTGTTCCGTTTGGTCAACTAAAGACCATGGACCAGAACTCATGGCAAGCACGTAATGAAGCCTTACAGTTCCGTAATGAGGCTGAGACCTTCAGAAACCAAGCGGAGGGCTTTAAGAACGAGTCCAGTACCAACGCTACGAACACAAAGCAGTGGCGCGATGAGACCAAGGGTTTCCGAGACGAAGCCGAGCGGTTCAAGAATACGGCTGGTCAATACGCTACATCTGCTGGGAACTCTGCTTCCGCTGCGCATACATCTGAGGTAAACGCTgctgtctcttt                                                                                                                                                                                                               >  1:372552/1‑291 (MQ=255)`  
                                                                                              `ccGTGGATGACCGCGATGCTGTTCCGTTTGGTCAACTAAAGACCATGGACCAGAACTCATGGCAAGCACGTAATGAAGCCTTACAGTTCAGTAATGAGGCTGAGACTTTCAGAAACCAAGCGGAGGGCTTTAAGAACGAGTCCAGTACCAACGCTACGAACACAAAGCAGTGGCGCGATGAGACCAAGGGTTTCCGAGACGAAGCCGAGCGGTTCAAGAATACGGCTGGTCAATACGCTACATCTGCTGGGAACTCTGCTTCCGCTGCGCATCAATCTGAGGTCAACGCTGAGAACTCTGc                                                                                                                                                                                                               >  1:319676/1‑301 (MQ=255)`  
                                                                                                        `ccGCGATGCTGTTCCGTTTGGTCAACTAAAGACCATGGACCAGAACTCATGGCAAGCACGTAATGAAGCCTTACAGTTCCGTAATGAGGCTGATACTTTCAGAAACCAAGCGGAGGGCTTTAAGAACGAGTCCAGTACCAACGCTACGAACACAAAGCAGTGGCGCGATGATACCAAGGGTTTCCGAGACGAAGCCGAGCGGTTCAAGAATACGGCTGGTCAATACGCTACATCTGCTGGGAACTCTGCTTCCGCTGCGCATCAATCTGAGGTAAACGCTGAGAACTCTGCCACAGCATcc                                                                                                                                                                                                     >  1:216659/1‑301 (MQ=255)`  
                                                                                                                `tgtgtataagagacagaCTAAAGACCATGGACCAGAACTCATGGCAAGCACGTAATGAAGCCTTACAGTTCCGTAATGAGTCTGAGACTTTCAGAAACCAAGCGGAGGGCTTTAAGAACGAGTCCAGTACCAACGCTACTAACACAAAGCAGTGGCGCGATGAGACCAAGGGTTTCCGAGACGAAGCCGAGCGGTTCAAGAATACGGCTGGTCAATACGCTACATCTGCTGGGAACTCTGCTTACGCTGCGCATCAATCTGAGGTAAACGCTGAGAACTCTGCCACAGCATCCGCTAActa                                                                                                                                                                                             <  1:427684/285‑2 (MQ=255)`  
                                                                                                                        `tgtgaataagggacagccATGGACCAGAACTCATGGCAAGCACGTAAGGAAGCCTTACAGTTCCGTAATGAGGCTGAGACTTTCAGAAACCAAGCGGAGGGCTTTAAGAACGAGTCCAGTACCAACGCTACGAACACAAAGCAGTGGCGCGATGAGACCAAGGGTTTCCGAGACGAAGCCGAGCGGTTCAAGAATACGGCTGGTCAATACGCTACATCTGCTGGGAACTCTGCTTCCGCTGCGCATCAATCTGAGGTAAACGCTGAGAACTCTGCCACAGCATCCGCTAACTATGCTCAtt                                                                                                                                                                                     <  1:387781/285‑1 (MQ=255)`  
                                                                                                                           `ggTCAACTAAAGACCATGGACCAGAACTCATGGCAAGCACGTAATGAAGCCTTACAGTTCCGTAATGAGGCTGAGACTTTCAGAAACCAAGCGGAGGGCTTTAAGAACGAGTCCAGTACCAACGCTACGAACACAAAGCAGTGGCGCGATGAGACAAAGGGTTTCCGAGACGAAGCCGAGCGGTTCAAGAATACGGCTGGTCAATACGCTACATCTGCTGGGAACTCTGCTTCAGCTGCGCATCAATCTGAGGTAAACGCTGAGAACTCTGCCACAGCATCCGCCAACTATGCTCAATTgg                                                                                                                                                                                  >  1:95633/1‑301 (MQ=255)`  
                                                                                                                                `aCTAAAGACCATGGACCAGAACTCATGGCAAGCACGTAATGAAGCCTTACAGTTCCGTAATGAGGCTGAGACTTTCAGAAACCAAGCGGAGGGCTTTAAGAACGAGTCCAGTACCAACGCTACGAACACAAAGCAGTGGCGCGATGAGACCAAGGGTTTCCGAGACGAAGCCGAGCGGTTCAAGAATACGGCTGGTCAATACGCTACATCTGCTGGGAACTCTGCTTCCGCTGCGCATCAATCTGAGGTAAACGCTGAGAACTCTGCCACAGCATCCGCTAAcctgtatcttatacacatc                                                                                                                                                                             >  1:48472/1‑283 (MQ=255)`  
                                                                                                                                `aCTAAAGACCATGGACCAGAACTCATGGCAAGCACGTAATGAAGCCTTACAGTTCCGTAATGAGGCTGAGACTTTCAGAAACCAAGCGGAGGGCTTTAAGAACGAGTCCAGTACCAACGCTACGAACACAAAGCAGTGGCGCGATGAGACCAAGGGTTTCCGAGACGAAGCCAAGCGGTTCAAGAATACGGCTGGTCAATACGCTACATCTGCTGGGAACTCTGCTTCCGCTGCGCATCAATCTGAGGTAAACGCTGAGAACTCTGCCACAGCATCCGCTAActactgtctcttctacacc                                                                                                                                                                             >  1:157868/1‑284 (MQ=255)`  
                                                                                                                                `aCTAAAGACCATGGACCAGAACTCATGGCAAGCACGTAATGAAGCCTTACAGTTCCGTAATGAGGCTGAGACTTTCAGAAACCAAGCGGAGGGCTTTAAGAACGAGTCCAGTACCAACGCTACGAACACAAAGCAGTGGCGCGATGAGACCAAGGGTTTCCGAGACGAAGCCAAGCGGTTCAAGAATACGGCTGGTCAATACGCTACATCTGCTGGGAACTCTGCTTCCGCTGCGCATCAATCTGAGGTAAACGCTGAGAACTCTGCCACAGCATCCGCTAACTATGCTCATTTGGcctgg                                                                                                                                                                             >  1:348675/1‑297 (MQ=255)`  
                                                                                                                                `aCTAAAGACCATGGACCAGAACTCATGGCAAGCACGTAATGAAGCCTTACAGTTCCGTAATGAGGCTGAGACTTTCAGAAACCAAGCGGAGGGAGTTAAGAACGAGTCCAGTACCAACGCTACGAACACAAAGCAGTGGCGCGATGAGACCGAGGGATTCCGAGACGAAGCCAAGCGGTTCAAGAATACGGCTGGTCAATACGCTACATCTGCTGGGAAATCTGCTTCCGCGGCGCATCAATCTGAGGTAAACGCTGAGAAGTCTGCCACAGCATCCGCTAACTATGATCATTGGGCAGaa                                                                                                                                                                             >  1:203973/1‑301 (MQ=255)`  
                                                                                                                                        `ccATGGACCAGAACTCATGGCAAGCACGTAATGAAGCCTTACAGTTCCGTAATGAGGCTGAGACTTTCAGAAACCAAGCGGAGGGCTTTAAGAACGAGTCCAGTACCAACGCTACGAACACAAAGCAGTGGCGCGATGAGACCAAGGGTTTCCGAGACGAAGCCAAGCGGTTCAAGAATATGGCTGGTCAATACGCTACATCTGCTGGGAACTCTGCTTCCGCTGCGCATCAATCTGAGGTAAACGCTGAGAACTCTGCCACAGCATCCGCTAACTCTGCTCATTTGGCAGAAccagtctc                                                                                                                                                                     >  1:305692/1‑294 (MQ=255)`  
                                                                                                                                                  `gAACTCATGGCAAGCACGTAATGAAGCCTTACAGTTCCGTAATGAGGCTGAGACTTTCAGAAACCAAGCGGAGGGCTTTAAGAACGCGTCCAGTACCAACGCTACGAACACAAAGCAGTGGCGCGATGAGACCAAGGGTTTCCGAGACGAAGCCAAGCGGTTCAAGAATACGGCTGGTCAATACGCTACATCTGCTGGGAACTCTGCTTCCGCTGCGCATCAATCTGAGGTAAACGCTGAGAACTCTGCCACAGCCTCCGCTAACTATGCTCCTTTGGCAGAACAGCAAGCCGACCGTACg                                                                                                                                                           >  1:353451/1‑301 (MQ=255)`  
                                                                                                                                                         `tGGCAAGCACGTAATGAAGCCTTACAGTTCCGTAATGAGGCTGAGACTTTCAGAAACCAAGCGGAGGGCTTTAAGAACGAGTCCAGTACCAACGCTACGAACACAAAGCAGTGGCGCGATGAGACCAAGGGTTTCCGAGACGAAGCCGAGCGGTTCAAGAATACGGCTGGTCAATACGCTACATCTGCTGGGAACTCTGCTTCCGCTGCGCATCAATCTGAGGTAAACGCTGAGAACTATGCCACAGCATCCGCTAACTATGCTCATTTGGCAGAACAGCAAGCAGACCGTGCGGAACGTg                                                                                                                                                    >  1:342543/1‑301 (MQ=255)`  
                                                                                                                                                            `tcggagatgtgtataagagacaggTTCCGTAATGAGGCTTAGACTTCCAGAAACCAAGCGGAGGGCTTTAAGAACGAGTCCAGTACCAACGCTACGAACACAAAGCAGTGGCGCGATGAGACCAAGGGTTTCCGAGACGAAGCCAAGCGGTTCAAGAATACGGCTGGTCAATACGCTACATCTGCTGGGAACTCTGCTTCCGCTGCGCATCAATCTGAGGTAAACGCTGAGAACTCTGCCACAGCATCCGCTAACTATGCTCATTTGGCAGAACAGCAAGCAGACCGTGCGGAACGTGAgg                                                                                                                                                 <  1:15047/278‑1 (MQ=255)`  
                                                                                                                                                                    `tAATGAAGCCTTACAGTTCCGTAATGAGGCTGAGACTTTCAGAAACCAAGCGGAGGTCTTTAAGAACGAGTCCAGTACCAACGCTACGAACACAAAGCAGTGGCGCGATGAGACCAAGGGTTTCCGAGACGAAGCCAAGCGGTTCAAGAATACGGCTGGTCAATACGCGACATCTGCTGGGAACTCTGCTTCCGCTGCGCATCAATCTGAGGTAAACGCTGAGAACTCTGCCACAGCATCCGCTAACTATGCTCATTTGGCAGCACCGCAAGCAGACCGTGCGGAACGTGAGGCAGCCAAg                                                                                                                                         >  1:276883/1‑301 (MQ=255)`  
                                                                                                                                                                    `tAATGAAGCCTTACAGTTCCGTAATGAGGCTGAGACTTTCAGAAACCAAGCGGAGGGCTTTAAGAACGAGTCCAGTACCAACGCTACGAACACAAAGCAGTGGCGCGATGAGACCAAGGGTTTCCGAGACGAAGCCGAGCGGTTCAAGAATACGGCTGGTCAATACGCTACATCTGCTGGGAACTCTGCTTCCGCTGCGCATCAATCTGAGGTAAACGCTGAGAACTCTGCCACAGCATCCGCTAACTCTGCTCATTTGGCAGAACAGCAAGCAGACCGTGCGGAACGTGAGGCAGACAAg                                                                                                                                         >  1:225474/1‑301 (MQ=255)`  
                                                                                                                                                                     `aaTGAAGCCTTACAGTTCCGTAATGAGGCTGAGACTTTCAGAAACCAAGCGGAGGGCTTTAAGAACGAGTCCAGTACCAACGCTACGAACACAAAGCAGTGGCGCGATGAGACCAAGGGTTTCCGAGACGAAGCCGAGCGGTTCAAGAATACGGCTGGTCAATACGCTACATCTGCTGGGAACTCTGCTTCCGCTGCGAATCAATCTGAGGTAAACGCTGAGAACTCTGCCACAGCATCCGCTAACTATGCTCATTTGGCAGAACAGCAAGCAGACCGTGCGGAACGTGAGGCAGACAAGc                                                                                                                                        >  1:300876/1‑301 (MQ=255)`  
                                                                                                                                                                        `gAAGCCTTACAGTTCCGTAATGAGGCTGAGACTTTCAGAAACCAAGCTGAGGGCTTTAAGAACGAGTCCAGTACCAACGCTACGAACACAAAGCAGTGGCGCGATGAGACCAAGGGTTTCCGAGACGAAGCCAAGCGGTTCAAGAATACGGCTGGTCAATACGCTACATCTGCTGGGAACTCTGCTTCCGCTGCGCATCAATCTGAGGTAAACGCTGAGAACTCTGCCACAGCATCCGCTAACTATGCTCATTTGGCAGAACAGCAAGCAGACCGTGCGCAACGTGAGGCAGACAAGCTgg                                                                                                                                     >  1:331791/1‑301 (MQ=255)`  
                                                                                                                                                                        `gAAGCCTTACAGTTCCGTAATAAGGCTGAGACTTTCAGAAACCAAGCGGAGGGCTTTAAGAACGAGTCCAGTACCAACGCTACGAACACAAAGCAGTGGCGCGATGAGACCAATGGTTTCCGAGACGAAGCCAAGCGGTTCAAGAATACGGCTGGTCAATACGCTACATCTGCTGGGAACTCTGCTTCCGCTGCGCATCAATCTGAGGTAAACGCTGAGAACTCTGCCACAGCATCCGCTAACTATGCTCATTTGGCAGAACAGCAAGCAGATCGTGCGGAACGTGAGGCAGACAAGCTgg                                                                                                                                     <  1:309308/301‑1 (MQ=255)`  
                                                                                                                                                                           `gCCTTATAGTTCCGTAATGAGGCTGAGACTTTCAGAAACCAAGCGGAGGGCTTTAAGAACGAGTCCAGTACCAACGCTACGAACACAAAGCAGTGGCGCGATGAGACCAAGAGTTTCCGAGACGAAGCCAAGCGGTTCAAGAATACGGCTGGTCAATACGCTACATCTGCTGGGCACTCTGCTTCCGCTGCGCATCAATCTGAGGTAAACGCTGAGAACTCTGCCACAGCATCCGCTAACTCTGCTCATTTGGCAGAACAGCAAGAAGACCGTGAGGAACGAGAGGAAGACactgtctctt                                                                                                                                  >  1:293700/1‑292 (MQ=255)`  
                                                                                                                                                                           `gCCTTACAGTTCCGTAATGAGGCTGAGACTTTCAGAAACCAAGCGGAGGGCTTTAAGAACGAGTCCAGTACCAACGCTACGAACACAAAGCAGTGGCGCGATGAGACCAAGGGTTTCCGAGACGAAGCCAAGCGGTTCAAGAATACGGCTGGTCAATACGCTACATCTGCTGGGAACTCTGCTTCCGCTGCGCATCAATCTGAGGTAAACGCTGAGAACTCTGCCACAGAATCCGCTAACTATGCTCATTTGGCAGAACAGCAAGCAGACCGTGCGGAACGTGAgctggctcttatacaca                                                                                                                                  >  1:493133/1‑285 (MQ=255)`  
                                                                                                                                                                           `gCCTTACAGTTCCGTAATGAGGCTGAGACTTTCAGAAACCAAGCGGAGGGCTTTAAGAACGAGTCCAGTACCAACGCTACGAACACAAAGCAGTGGCGCGATGAGACCAAGGGTTTCCGAGACGAAGCCAAGCGGTTCAAGAATACGGCTGGGCAATACGCTCCATCTTCTGGGAACTCTTCTTCCGCTGCGCATCAATCTGAGTTAAACGCTGAGAACTCTGCCACAGCATCCGCTAACTATGCTCATTTGGCAGAACAGCAAGCAGACCGTTCGTCACGTGAGTCAGACAAGCAGGaaa                                                                                                                                  >  1:106636/1‑301 (MQ=255)`  
                                                                                                                                                                                     `tCCGTAATGAGGCTGAGACTTTCAGGAACCAAGCGGAGGGCTTTAAGAACGAGTCCAGTACCAACGCTACGAACACCAAGCAGTGGCGCGATGAGACCAAGGATTTCCGAGACGAAGCCGAGCGGTTCAAGAATACGGCTGGTCAATACGCTACATCTGCTGGGAACTCTGCTTCCGCTGCGCATCAATCTGAGGTAAACGCTTAGAACTCTGCCACATCATCCGCCAACTCTGCTCATTTGTCAGAACAGCAAGCAGACAGTGCGCAAGCTGAGGCAGACAAGCTGGAAACTTACAATgg                                                                                                                        >  1:260561/1‑301 (MQ=255)`  
                                                                                                                                                                                     `tCCGTAATGAGGCTGAGACTTTCAGAAACCAAGCGGAGGGCTTTAAGAACGAGTCCAGTACCAACGCTACGAACACAAAGCAGTGGCGCGATGAGACCAAGGGTTTCCGAGACGAAGCCGAGCGGTTCAAGAATACGGCTGGTCAATACGCTACATCTGCTGGGAACTCTGCTTCCGCTGCGCATCAATCTGAGGTAAACGCTGAGAACTCTGCCACAGCATCCGCTAACTATGCTCATTTGGCAGAACAGACAGCAGACCGTGCGGAACGTGGGGCAGACAAGCTGGAGAATTACaaacg                                                                                                                        >  1:297544/1‑298 (MQ=255)`  
                                                                                                                                                                                     `tCCGTAATGAGGCTGAGACTTTCAGAAACCAAGCGGAGGGCTTTAAGAACGAGTCCAGTACCAACGCTACGAACACAAAGCAGTGGCGCGATGAGACCAAGGGTTTCCGAGACGAAGCCAAGCGGTTCAAGAATATGGCTGGTCAATACGCTACATCTGCTGGGAACTCTGCTTCCGCTGCGCATCAATCTGAGGTAAACGCTGAGAACTCTGCCACAGCATCCGCTAACTATGCTCATTTGGCAGAACAGCAAGCAGACCGTGCGGAACGTGAGGCAGACAAGCTGGAAAATTACAATgg                                                                                                                        >  1:82956/1‑301 (MQ=255)`  
                                                                                                                                                                                      `ccGTAATGAGGCTGAGACTTTCAGAAACCAAGCGGAGGGCTTTAAGAACGAGTCCAGTACCAACGCTACGAACACAAAGCAGTGGCGCGATGAGACCAAGGGTTTCCGAGACGAAGCCAAGCGGTTCAAGAATACGGCTGGTCAATACGCTACATCTGCTGGGAACTCTGCTTCCACTGCGCATCAATCTGAGGTAAACGCTGAGAACTATGCCACAGCATCCGCTAACTATGCTCCTTTGGCAGAACAGCAAGCAGACCGTCCGGAACGTGCGGCAGACAACCTGGAAAATTACATTGGa                                                                                                                       >  1:473447/1‑301 (MQ=255)`  
                                                                                                                                                                                        `gTAATGAGTCCGAGACTTTCAGAAACCAAGCGGAGGGCGTTAAGAACGAGTCCAGTACCAACGCTACGAACACAAAGAAGTGGCGCGATGAGACCAAGGGTTGCAGAGACGAAGCTGAGCGGTTCAAGAATACGGCTGGTCAATACGCTACATCTGCTGGGAACTCTGCTTCCGCTGCGCATCAAACTGAGGTAAACGCTGAGAACTCTGCCACAGCATCCGCTAACTATGCACATTTGGCAGAACAGCAAGCAGACCGTGCGGAACGTGAGGAAGACAAGCTGGAAAATTACAATGGAtt                                                                                                                     <  1:335905/301‑1 (MQ=255)`  
                                                                                                                                                                                           `aTGAGGCTGATACTTTCAGAAACCAAGCTGAGGGCTTTAAGAACGAGTCCAGTACCAACGCTACGAACACAAAGCATTGGCGCGATGAGACCTAGTGTTTCCGAGACGAAGCCGAGCGGTTCAAGAATACGGCTGGTCAATACGCTACATCTGCTGGGAACTCTGCTTCCGCTGCGCATCAATCTGAGGTAAACGCTGAGAACTCTGCCACAGCATCCGCTAACTATGCTCATTTGGCAGAACAGCAAGCAGACCGTGCGGAACGTGAGGCAGACAAGATGGAAAATTACAATGGATTGGc                                                                                                                  <  1:383118/301‑1 (MQ=255)`  
                                                                                                                                                                                            `tGAAGCTGGGACTTTCAGAAACCAAGCGGAGGGCTTTAAGAACGAGTCCAGTACCAACGCTACGAACGCAAAGCAGTGGCGCGATGAGACCAAGGGTTTCCGAGACGAAGCCGAGCGGTTCAAGAATACGGCTGGTCAATACGCTACATCTGCTGGGAACTCTGCTTCCGCTGCGAATCAATCTGAGGTAAACGCTGAGAACTCTGCCACAGCATCCGCTAACTATGCTCATTTGGCAGAACAGCAAGCAGACCGTGCGGAACGTGAGGCAGACAAGCTGGAAAATTACAATGGATTGGCt                                                                                                                 <  1:168485/301‑1 (MQ=255)`  
                                                                                                                                                                                                 `ttGAGACTTTCAGAAGCCAAGCGGAGGGCTTTAAGAACGAGTCCAGTACCAACGCTACGAACACAAAGAAGTGGCGCGATGAGACCAAGGGTTTCCGAGACGAAGCCAAGCGGTGCAAGAATACGGCTGGTCAATACGCTACATCTGCTGGGAACTCTGCTTCTGCTGCGCATCAATCTGCGGTAAACGCTGAGAACTCTGCCACAGCATCCGCTAACTATGCTCATTTGGCAGAACAGCAAGCAGACCGTGCGGAACGTGAGGCAGACAAGCTGGAAAATTACAATGGATTGGCTGGTGc                                                                                                            <  1:230667/300‑1 (MQ=255)`  
                                                                                                                                                                                                      `aCTTACAGAAACAAAGCGGAGCGTGGTAAGAACGAGGCAAGTACAGACGCTACGAACAAAAAGCAGGGGCGAGATGAGACCAAGGGTTGACGAGACGAAGCCGAGCGGTGCAATAATACGGCTGGTCAAGACGTTACATCGGCTGGGAACTCTGCTTCCGCTGCGCATCAATCGGAGGTAAACGCTGAGAACTCTGCCACAGCATCAGCGAACTATGCTCATTTGGCAGAACAGCAAGCAGACCGTGCGGAACGTGAGGCAGACAAGCTGGAAAATTACAATGGATTGGCTGGTGCAATTg                                                                                                       <  1:326121/301‑1 (MQ=255)`  
                                                                                                                                                                                                         `ttCAGAAACCAAGCGGAGGGCTTTAAGAACGAGTCCAGTACCAACGCTACGAACACAAAGCAGTGGCGCGATGAGACCAAGGGTTTCCGAGACGAATCCGAGCGGTTCAAGAATACGGCTTGTCAATACGCTACCTCTGCTGGGAACTCTGCTTCCGCTGCGCATCAATCTTAGGTAAACGCTGCGAACTCTGCCACAGCATCCGCTCACTATGCTCCTTTTGCAGAACAGCAAGCAGACCGTTCTGAACGTGAGGCAGACACGCTTGAAAATTACAATGGATTGGCTGGTGCAATTGAtt                                                                                                    >  1:401680/1‑300 (MQ=255)`  
                                                                                                                                                                                                                `aCCAAGCGGAGGGCTTTAAGAACGAGTCCAGTACCAACGCTACGAACACAAAGCAGTGGCGCGATGAGACCAAGGGTTTCCGAGACGAAGCCAAGCGGTTCAAGAATACGGCTGGTCAATACGCTACATCTGCTGGGAACTCTGCTTCCGCTGCGCATCAATCTGAGGTAAACGCTGAGAACTCTGCCACAGCATCCGCTAACTATGCTCATTTGGCAGAACAGCAAGCAGACCGTGCGGAACGTGAGGCAGAAAAGCTGGAAAATTACAATGGATAGGCTGGCGAACTTGATAAGGtctg                                                                                             >  1:450626/1‑298 (MQ=255)`  
                                                                                                                                                                                                                 `ccAAGCGGCGGGCTTTAAGAACGAGTCCAGTACCAACGCTACGAACACAAAGCAGTGGCGCGATGAGACCAAGGGTTTCCGAGACGAAGCCAAGCGGTTCAAGAATATGGCTGGTCAATACGCTACATCTGCTGGGAACTCTGCTTCCGCTGCGCATCAATCTGAGGTAAACGCTGAGAACTCTGCCACAGCATCCGCTAACTATGCTCATTTGGCAGAACAGCAAGCAGACCGTTCGGAACGTGAGGCAGACAAGCTGGAAAATTACAATGGATTGGCTGGTGCAATTGATAAGGTAGAt                                                                                            >  1:465275/1‑301 (MQ=255)`  
                                                                                                                                                                                                                 `ccAAGCGGAGGGCTTTAAGAACGAGTCCAGTACCAACGCTACGAACACAAAGCAGTGGCGCGATGAGACCAAGGGTTTCCGAGACGAAGCCGAGCGGTTCAAGAATACGGCTGGTCAATACGCTACATCTGCTGGGAACTCTGCTTCCGCTGCGCATCAATCTGAGGTAAACGCTGAGAACTCTGCCACAGCATCCGCTAACTATGCTCATTTGGCAGACCAGCAAGCAGACAGTGCGGAACGTGAGGCAGAACAGCTGGACACTGACAATGGATTGGCTTGAGCCATTGATAAGGTAGAt                                                                                            >  1:489105/1‑301 (MQ=255)`  
                                                                                                                                                                                                                   `aaGCGGAGGGCTTTAAGAACGAGTCCAGTACCAACGCTACGAACACAAAGCAGTGGCGCGATGAGACCAAGGGTTTCCGAGACGAAGCCAAGCGGTTCAAGAATACGGCTGGTCAATACGCTACATCTGCTGGGAACTCTGCTTCCGCTGCGCATCAATCTGAGGTAAACGCTGAGAACTCTGCCACAGCATCCGCTAACTATGCTCATTTGGCAGAACAGCAAGCAGACCGTGCGGAACGTGAGGCAGACAAGCTGGAAAATTACAATGGATTGGCTGGTGCAATTGATAAGGTAGATgg                                                                                          <  1:469672/301‑1 (MQ=255)`  
                                                                                                                                                                                                                           `ggCTTTAAGAACGAGTCCAGTACCAACGCTACGAACACAAAGCAGTGGCGCGATGAGACCAAGGGTTTCCGAGACGAAGCCAAGCGGTTCAAGAATACGGCTGGTCAATACGCTACATCTGCTGGGAACTCTGCTTCCGCTGCGCATCAATCTGAGGTAAACGCTGAGAACTCTGCCACAGCATCCACTAACTATGCTCATTTGGCAGAACAGCACGCAGACCGTGCGGAACGTGAGGCAGACAAGCTGGAAAATTACAATGGATTGGCTGGTGCAATTGATAAGGTAGATGGAACCAAtg                                                                                  <  1:460614/301‑1 (MQ=255)`  
                                                                                                                                                                                                                                `tAAGAACGAGTCCAGTACCAACGCTACCAACACAAAGCAGTGGCGCGATGAGACCCAGGGTTTACGAGACGAAGCCAAGCGGGTCAAGAATACGGCTGGTCAAGACGTTACATCGGCGGGGAACTCTGTTTCCGCTGCGCATCAATATGAGGTAAACGCTGAGACCTCTGCCACAGCATCCGCTAACTATGCTCATTTGGCAGAACAGCAAGCAGACCGTGCGGAACGTGAGGCAGACAAGCTGGAAAATTACAATGGATTGGCTGGTGCAATTGATAAGGTAGATGGAACCGATGTGTAc                                                                             <  1:367443/301‑1 (MQ=255)`  
                                                                                                                                                                                                                                    `aaCGAGTCCAGTACCAACGCTACGAACACAAAGCAGTGGCGCGATGAGACCAAGGGTTTCCGAGACGAAGCCAAGCGGTTCAAGAATACGGCTGGTCAATACGCTACATCTGCTTGGAACTCTGCTTCCGCTGCGCATCAATCTGAGGTAAACGCTGAGAACTCTGCCACAGCATCCGCTAACTATGCTCATTTGGCAGAACAGCAAGCAGACCGTGCGGAACGTGAGGCAGACAAGCTGGAAAATTACAATGGATTGGCTGGTGCAATTGATAAGGTAGATGGAACCAATGTGTACTgga                                                                         >  1:177435/1‑301 (MQ=255)`  
                                                                                                                                                                                                                                        `ggTCCAGTACCAACGCTACGAACACAAAGCAGTGGCGCGATGAGACCAAGGGTTTCCGAGACGAAGCCAAGCGGTTCAAGAATACGGCTGGTCAATACGCTACATCTGCTGGGAACTCTGCTTCAGCTGCGCATCAATCTGAGGTAATCGCTGAGAACTATGCCACAGCATCCGCTAACTATGCTCATTTGGCAGAACAGCAAGCAGACCGTGCGGAACGTGAGGCAGACAAGCTGGAAAATTACAATGGATTGGCTGGTGCAATTGATAAGGTAGATGGAACCAATGTGTACTggaaagg                                                                     <  1:406395/300‑1 (MQ=255)`  
                                                                                                                                                                                                                                         `gTCCAGTACCAACGCTACGAACACAAAGCAGTGGCGCGATGAGACCAATGGTTTCCGAGACGAAGCCGAGCGGTTCAAGAATACGGCTGGTCAATACGCTACATCTGCTGGGAACTCTGCTTCCGCTGCGCATCAATCTGAGGTAAACGCTGAGAACTCTGCCACAGCATCCGCTAACTATGCTCATTTGGCAGAACAGCAAGCAGACCGTGCGGAACGTGAGGCAGACAAGCTGGAAAATTACAATGGATTGGCTGGTGCAATTGATAAGGTAGATGGAACCAATGTGTACTggaaagga                                                                    >  1:249535/1‑301 (MQ=255)`  
                                                                                                                                                                                                                                         `gTCCAGTACCAACGCTACGAACACAAAGCAGTGGCGCGATGAGACCAAGGGTTTCCGAGACGAAGCCAAGCGGTTAAAGAATACGGCTGGTCAATACGCTACATCTGCTGGGAACTCTGCTTCCGCTGCGCATCAATCTGAGGTAAACGCTGAGAACTCTGCCACAGCATCCGCTAACTATGCTCATTTGGCAGAACAGCAAGCAGACCGTGCGGAACGTGAGGCAGACAAGCTGGAAAATTACAATGGATTGGCTGGTGCAATTGATAAGGTAGATGGAACCAATGTGTACTggaaagta                                                                    >  1:181748/1‑299 (MQ=255)`  
                                                                                                                                                                                                                                          `tCCAGTACCAACGCTACGAACACAAAGCAGTGGCGCGATGAGACCAAGGGTTTCCGAGATGAAGCCGAGCGGTTCAAGAATACGGCTGGTCAATACGCTACATCTGCTGGGAACTCTGCTTCCGCTGCGCATCAATCTGAGGTAAACGCTGAGAACTCTGCCACAGCATCCGCTAACTATACTCATTTGGCAGAACAGCAAGCAGACCGTGCGGAACGTGAGGCAGACAAGCTGGAAAATTACAATGGATTGGCTGGTGCAATTGATAAGGTAGATGGAAACAATGTGTAcctgtctctta                                                                   >  1:124819/1‑291 (MQ=255)`  
                                                                                                                                                                                                                                           `ccAGTACCAACGCTACGAACACAAAGAAGTGGCGCGATGAGACCAAGCGTTTCCGAGACGAAGCCAAGCGGTTCAAGAATACGGCTTGTCAATACGCTACATCTGCTGGGAACTCTGCTTCCGCTGCGCATCAATCTGAGGTAAACGCTGAGAACTCTGCCACAGCATCCGCTAACTATGCTCATTTGGCAGAACAGCAAGCAGACCGTGCGGAACGTGAGGCAGACAAGCTGGAAAATTACAATGGATTGGCTGGTGCAATTGATAAGGTAGATGGAACCAATGTGTACTggaaaggaaa                                                                  <  1:13804/301‑1 (MQ=255)`  
                                                                                                                                                                                                                                              `gTACCAACGCTACGAACACAAAGCAGTGGCGCGATGAGACCAAGGGTTTCCGAGACGAAGCCGAGCGGTTCAAGAATACGGCTGGTCAATACGCTACATCTGCTGGGAACTCTGCTTCCGCTGCGCATCAATCTGAGGTAAACGCTGATAACTCTGCCACGGCATCCGCTAACTCTGCTCATTTGGCAGAACAGCAAGCAGACCGTGCGGAACGTGAGGCAGACAAGCTGGAAAATTACAATGGATTGGCTGGTGCAATTGATAAGGTAGATGGAACCGAtgctgtctcttatacacatct                                                               >  1:189953/1‑282 (MQ=255)`  
                                                                                                                                                                                                                                              `gTACCAACGCTACGAACACAAAGCAGTGGCGCGATGAGACCAAGGGTTTCCGAGACGAAGCCAAGCGGTTCAAGAATACGGCTGGTCAATACGCTACATCTGCTTGGAACTCTGCTTCCGCTGCGCATCAATCTGAGGTAAACGCTGAGAACTCTGCCACAGCATCCGCTAACTATGCTCATTTGGCAGAACAGCAAGCAGACCGTGCGGAACGTGAGGCAGACAAGCTGGAAAATTACAATGGATTGGCTGGTGCAATTGATAAGGTAGATGGAACCAATGTGTACTGGAAAGGAAatat                                                               >  1:265377/1‑301 (MQ=255)`  
                                                                                                                                                                                                                                                       `cTACGAACACAAAGCAGTGGCGCGATGAGACCAAGGGTTTCCGAGACGAAGCCAAGCGGTTCAAGAATACGGCTGGTCAATACGCTACATCTGCTGGGAACTCTGCTTCCGCTGCGCATCAATCTGAGGTAAACGCTGAGAACTCTGCCACAGCATCCGCTAACTATGCTCATTTGGCAGAACAGCAAGCAGACCGTGCGGAACGTGAGGAAGACAAGCTGGAAAATTACAATGGATTGGCTGGTGCAATTGATAAGGTAGATGGAACCAATGTGTACTGGAAAGGAAATATTCACGCTaa                                                      >  1:287806/1‑301 (MQ=255)`  
                                                                                                                                                                                                                                                         `aCGAACACAAAGCAGTGGCGCGATGAGACCAAGGGTTTCCGAGACGAAGCCAAGCGGTTCAATAATCCGGCTGGTCAATACGCTACATCTGCTGGGAACTCTGCTTCCGCTGCGCATCAATCTGAGGTAAACGCTGAGACCTCTGCCACAGCATCCGCTAACTATGCTCATTTGGCGAAACAGCCAGCAGACCGTGCGGAACGTGAGGCAGACAAGCTGGAAAATTACAATTGAATGGCTGGTGCAATTGATAAGGTAGATGGAACCAATGTGTACTGGAAAGGAAATATTCACGATAACg                                                    >  1:503724/1‑301 (MQ=255)`  
                                                                                                                                                                                                                                                               `acaAAGCCGTGGCGCGATGAGACCAAGGGTTTCCGAGACGAAGCCGAGCTGTTCAAGAATACGGCTGGTCAATACGCTACATCTGCTGGGAACTCTGCTTCCGCTGCGCATCAATCTGAGGTAAACGCTGAGAACTCTGCCACAGCATCCGCTAACTATGCTCATTTGGCAGAACAGCAAGCAGACCGTGCGGAACGTGAGGCAGACAAGCTGGAAAATTACAATGGATTGGCTGGTGCAATTGATAAGGTAGATGGAACCAATGTGTACTGGAAAGGAAATATTCACGCTAACGGGCGcc                                              <  1:10403/301‑1 (MQ=255)`  
                                                                                                                                                                                                                                                               `acaAAGCAGTGGCGCGATGAGACCAAGGGTTTCCGAGACGAAGCCAAGCGGTTCAAGAATACAGCTGGTCAATACGCTACATCTGCTGGGAACTCTGCTTCCGCTGCGCATCAATCTGAGGTAAACGCTGAGAACTCTGCCACAGCATCAGCTAACTATGCTCATTTGGCAGAACAGCAAGCAGACCGTGCGGAACGTGAGGCAGACAAGCTGGAAAATTACAATGGATTGGCTGGTGCAATTGATAAGGTAGATGGAACCAATGTGTACTGGAAAGGAAATATTCACGCTAACGGGCGcc                                              <  1:185348/301‑1 (MQ=255)`  
                                                                                                                                                                                                                                                                  `aaGCAGTGGCGCGATGAGACCAAGGGTTTCCGAGACGAAG‑CAAGCGGTTCAAGAATACGGCTGGTCAATACGCTACATCTGCTGGGAACTCTGCTTCCGCTGCGCATCAATCTGAGGTAAACGCTGAGAACTATGCCACAGCATCCGCTAACTATGCTCATTTGGCAGAACAGCAAGCAGACCGTGCGGAACGTGAGGCAGACAAGCTGGAAAATTACAATGGATTGGCTGGTGCAATTGATAAGGTAGATGGAACCAATGTGTACTGGAAAGGAAATATTCACGCTAACGGGCGCCtttt                                          >  1:153183/1‑300 (MQ=255)`  
                                                                                                                                                                                                                                                                       `gTGGCGCGATGAGACCAAGGGTTTCCTAGATGAAGCCAAGCGGTTCAAGAATATGGCTGGTCAATACGCTACATCTGCTGGGAACTCTGCTTCCGCTGCGCATCAATCTGAGGTAAACGCTGAGAACTCTGCCACAGCATCCGCTAACTATGCTCATTTGGCCGAACAGCAAGCAGACCGTGCGGCACGTGAGGCAGACAAGCTGGAAAATTACAATGGATTGGCTGGTGCCATTGATAAGGTAGATGGAACCAATGTGTACTGGAAAGGCAATAGTCACGCTAACGGGCGCCTTTACATg                                      >  1:419442/1‑301 (MQ=255)`  
                                                                                                                                                                                                                                                                                     `ccAAGGGTTTCCGAGACGAAGCCAAGCGGTTCAAGAATACGGCTGGTCAATACGCTACATCTGCTGGGAACTCTGCTTCCGCTGCGCATCAATCTGAGGTAAACGCTGAGAACTCTGCCACAGCATCCGCTAACTATGCTCATTTGGCAGAACAGCAAGCAGACCGTGCGGAACGTGAGGCAGACAAGCTGGAAAATTACAATGGATTGGCTGGTGCAATTGATAAGGTAGATGGAACCAATGTGTACTGGAAAGGAAATATTCACGCTAACGGGCGCCTTTACATGAcctgtctcttata                        >  1:11778/1‑290 (MQ=255)`  
                                                                                                                                                                                                                                                                                              `tCCGAGACGAAGCCGAGCGGTTCAAGAATACGGCTGGTCAATACGATACATCTGCTGGGAACTCTGCTTCCGATGCGCATCAATCTGAGGTAAACGCTGAGAACTCTGCCACAGCATCCGCTAACTATGCTCATTTGGCAGAACAGCAAGCAGACCGTGCGGAACGTGAGGCAGACAAGCTGGAAAATTACAATGGATTGGCTGGTGCAATTGATAAGGTAGATGGAACCAATGTGTACTGGAAAGGAAATATTCACGCTAACGGGCGCCTTTACATGACCACAAACGGTTTTGACTGTgg               <  1:23905/301‑1 (MQ=255)`  
                                                                                                                                                                                                                                                                                                  `agaCGAAGCCGAGCGGTTCAAGAATACGGCTGGTCAATACGCTACATCTGCTGGGAACTCTGCTTCCGCTGCGCATCAATCTGAGGTAAACGCTGAGAACTCTGCCACAGCATCCGCTAACTATGCTCATTTGGCAGAACAGCAAGCAGACCGTGCGGAACGTGAGGCAGACAAGCTGGAAAATTACAATGGATTGGCTGGTGCAATTGATAAGGTAGATGGAACCAATGTGTACTGGAAAGGAAATATTCACGCTAACGGGCGCCTTTACATGACCACAAACGGTTTTGACTGTGGCCAg           >  1:411591/1‑301 (MQ=255)`  
                                                                                                                                                                                                                                                                                                      `aaaGCCAAGCGGTTCAAGAATACGGCTGGTCAATACGCTACATCTGCTGGGAACTCTGCTTCCGCTGCGCATCAATCTGAGGTAAACGCTGAGAACTCTGCCACAGCATCCGCTAACTATGCTCATTTGGCAGAACAGCAAGCAGACCGTGCGGAACGTGAGGCAGACAAGCTGGAAAATTACAATGGATTGGCTGGTGCAATTGATAAGGTAGATGGAACCAATGTGTACTGGAAAGGAAATATTCACGCTAACGGGCGCCTTTACCTGACCACAAACGGTTTTGACTGTGGCCAGTATc       <  1:432476/300‑1 (MQ=255)`  
                                                                                                                                                                                                                                                                                                         `gCCAAGCGGGTCAAGAATATGGCTTGTCAATACGCTACATCTGCTGGTAACTCTGCTTCCGCTGCGCATCAATCTGAGGTAAACGCTGAGAACTCTGCCACAGCATCCGCTAACTATGCTCATTTGGCAGAACAGCAAGCAGACCGTGCGGAACGTGAGGCAGACAAGCTGGAAAATTACAATGGATTGGCTGGTGCAATTGATAAGGTAGATTGAACCAATGTGTACTGGAAAGGAAATATTCACGCTAACGGGCGCCTTTACATGACCACAAACGGTTTTGACTGTGGCCAGTATCAAc    <  1:3976/301‑1 (MQ=255)`  

                                                                                                                                                                                                                                                                                                            `|`                                                                                                                                                                                                                                                                                                             
`GAAGAGGCCCGTGACCTCACTACGGATACTATCGGTGTCAATAACGATGGTCACTTGGATGCTCGTGGTCGTCGAATTGTGAACCTAGCGAACGCCGTGGATGACCGCGATGCTGTTCCGTTTGGTCAACTAAAGACCATGAACCAGAACTCATGGCAAGCACGTAATGAAGCCTTACAGTTCCGTAATGAGGCTGAGACTTTCAGAAACCAAGCGGAGGGCTTTAAGAACGAGTCCAGTACCAACGCTACGAACACAAAGCAGTGGCGCGATGAGACCAAGGGTTTCCGAGACGAAGCCAAGCGGTTCAAGAATACGGCTGGTCAATACGCTACATCTGCTGGGAACTCTGCTTCCGCTGCGCATCAATCTGAGGTAAACGCTGAGAACTCTGCCACAGCATCCGCTAACTCTGCTCATTTGGCAGAACAGCAAGCAGACCGTGCGGAACGTGAGGCAGACAAGCTGGAAAATTACAATGGATTGGCTGGTGCAATTGATAAGGTAGATGGAACCAATGTGTACTGGAAAGGAAATATTCACGCTAACGGGCGCCTTTACATGACCACAAACGGTTTTGACTGTGGCCAGTATCAACAG  >  NC_001604.1/34954‑35553`

  

Alignment Legend

Aligned base mismatch/match (shaded by quality score): `ATCG/ATCG` `< 3 ≤` `ATCG/ATCG` `< 9 ≤` `ATCG/ATCG` `< 19 ≤` `ATCG/ATCG` `< 33 ≤` `ATCG/ATCG` `< 38 ≤` `ATCG/ATCG`

Unaligned base: `atcg`    Masked matching base: `atcg`    Alignment gap: `‑`    Deleted base: `‑`
